# Identification and pharmaceutical evaluation of novel frog skin-derived serine proteinase inhibitor peptide–PE-BBI (*Pelophylax esculentus* Bowman-Birk inhibitor) for the potential treatment of cancer

**DOI:** 10.1038/s41598-018-32947-5

**Published:** 2018-09-28

**Authors:** Peng Lyu, Lilin Ge, Rui Ma, Ran Wei, Cian M. McCrudden, Tianbao Chen, Chris Shaw, Hang Fai Kwok

**Affiliations:** 1 Cancer Centre, Faculty of Health Sciences, University of Macau, Avenida de Universidade, Taipa, Macau SAR China; 2 Jiangsu Key Laboratory for Functional Substance of Chinese Medicine, Nanjing University of Chinese Medicine, 138 Xianlin Avenue, Qixia District, Nanjing, 215400 China; 3 Natural Drug Discovery Group, School of Pharmacy, Queen’s University Belfast, Belfast, BT9 7BL Northern Ireland UK

## Abstract

Amphibian venom-derived peptides have high potential in the field of anticancer drug discovery. We have isolated a novel Bowman-Birk proteinase inhibitor (BBI)-type peptide from the skin secretion of *Pelophylax esculentus* (PE) named PE-BBI, and evaluated its bio-functions and anti-cancer activity *in vitro*. PE-BBI is a heptadecapeptide with C-terminal amidation. The mRNA sequence and primary structure of PE-BBI were identified using RT-PCR and LC/MS, respectively. A trypsin inhibitory assay was used to characterize the serine proteinase inhibitory activity of synthetic PE-BBI. PE-BBI’s myotropic activity was analyzed using isolated rat bladder and rat-tail artery smooth muscle tissues, and the anti-cancer ability of PE-BBI using human colorectal cancer cells. PE-BBI’s mechanism of action was investigated using Discovery studio software. PE-BBI showed trypsin inhibitory activity (K_i_ = 310 ± 72 nM), strong myotropic activity, and cytotoxicity that were specific to cancer cells, and no side effect to normal epithelial cells. The docking stimulation showed that PE-BBI had high affinity to several members of human kallikrein related peptidase (KLK) family. This finding helps to enrich our understanding of BBI peptides’ mode of action. Moreover, the data presented here validates frog secretions as sources of potential novel proteinase inhibitors for cancer treatment.

## Introduction

The serine protease inhibitor family is one of the most widely distributed natural proteinase inhibitors. Serine protease inhibitors are able to regulate certain proteases participated in many human physiological and pathological processes, such as peptide hormone release, digestion, host defence, blood coagulation, disease progression, hypertension, muscular dystrophy, cancer, acquired immunodeficiency syndrome (AIDS), and microbial infections^1–4^. Serine proteinase inhibitors are also crucial in proteolytic activity disorder processes, which may cause a disruption in physiological processes, homeostatic balance, the expanding of tumour cells and degenerative diseases^5–7^. Understanding the roles serine protease inhibitors play in diseases may offer opportunities for development of therapeutic agents^8^. Proteinase inhibitors have been classified into more than 10 families, based on their structural features, including Kunitz, Kazal, and Bowman-Birk proteinase inhibitor (BBI) motifs^9^.

The BBI inhibitor family is a typical serine proteinase family, is commonly/largely found in the seeds of leguminous plants, and possesses strong trypsin and chymotrypsin inhibitory activities^10^. BBIs inhibit their targeted enzymes by a substrate-like manner through exposing their canonical conformation binding loop^11–14^. The two highly conserved canonical disulphide loops of BBI are the crucial structure for their trypsin and chymotrypsin inhibitory activities. In each loop, a hyper-exposed amino acid residue (lysine or arginine) called P_1_ residue (named by Schechter and Berger^15^) interacts with the active site of the enzyme, which is the most comment inhibitor-enzyme contacts and is responsible for inhibitor’s specificity^16^. Activated BBI can specifically bind to its correlated enzyme in a substrate-like manner, after which, the disulphide loop forms a stable complex with the active site of the proteinase^9,17^. The studies of BBIs have attracted increasing interest in the past decade due to their unique multiple functions. Within these studies, the functions of BBIs are ranging from pest control in transgenic plants, to the clinical aspect including anti-cancer, inflammatory, and allergic disorders^18^. In April 1992, BBI concentrate (BBIC), a soybean extract rich in BBI, have achieved Investigational New Drug status from the US Food and Drug Administration (FDA)^19^. BG-4, a BBI-type peptide was identified that has apoptosis-inducing activity in HCT-116 and HT-29 human colon cancer cell lines^20^. Recently, SFTI-1 (Sunflower trypsin inhibitor 1) and its structural modified variants showed specifically inhibition of kallikrein related peptidase (KLK) family. So far, some members of kallikrein family have been considered to be the diagnostic/prognostic biomarkers in different cancer types, including breast, ovarian, prostate and testicular carcinomas^21,22^. Furthermore, overexpression of KLK6, KLK8, and KLK10 were reported in colon cancer (CRC)^23^. Ogawa^24^ also further confirmed KLK6 mRNA overexpression leads to a poor prognosis in CRC patient cohorts.

Increasing evidence implies that BBI protease inhibitors were capable to inhibit the activity of KLK protein family, which are poor prognosis biomarkers for several carcinomas and other diseases reported in many studies^21–24^. As we know, cancer proliferation, migration, and invasion are complex pathological processes in which proteolytic enzymes were involved. As the expression and activity of these proteolytic enzymes have a strong correlation with the invasiveness and metastasis of cancer cells^25^, BBIs are being investigated as potential anti-colon cancer therapy. Here, we report the BBI-like peptide with anticancer activity found in frog skin secretion. PE-BBI (*Pelophylax esculentus* Bowman-Birk inhibitor) appeared to be a heptadecapeptide with C-terminal amidation. The molecular weight of PE-BBI (1.8 kDa) is much smaller than the canonical BBI protein (8~16 kDa) from legumina. PE-BBI contains the conserved disulphide loop between Cys^6^ and Cys^16^, which is homologous to the much bigger BBI family members. The synthetic replicates of PE-BBI were characterized by multiple organ-bath based *ex vivo* rat smooth muscle tissue probes and *in vitro* anticancer activity screening. We showed that PE-BBI is able to inhibit proliferation of several colon cancer cells without any obvious cytotoxicity to normal epithelial cells. These results suggested that PE-BBI might be a potential anticancer peptide, resultant of its protease inhibitory effects.

## Results

### Structural Characterisation of PE-BBI

**Figure 1:**
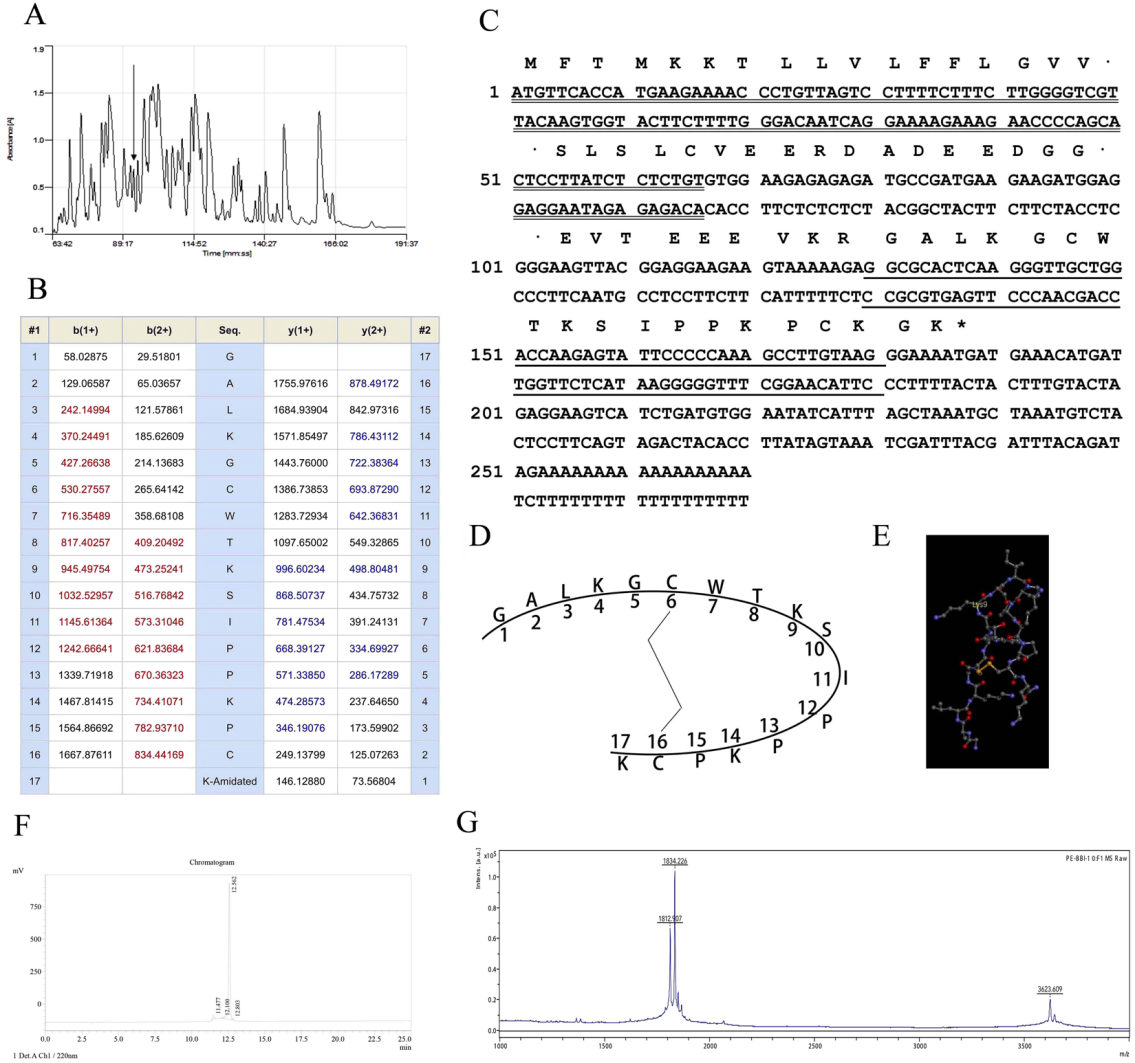
(**A**) Reverse phase HPLC chromatogram of lyophilised *Pelophylax esculentus* skin secretion, the arrow indicates the peak of PE-BBI. (**B**) Table of predicted mass of PE-BBI fragmentations with singly and doubly charged. The fractions detected by MS/MS were present in red and blue. (**C**) Nucleotide sequence of cDNA encoding the PE-BBI clone from *Pelophylax esculentus*. The putative signal peptide was indicated by double-underline. The mature peptide was labelled by single-underline. The stop codon (asterisk) was also indicated. (**D**) Schematic depiction of PE-BBI peptide. (**E**) Structure of PE-BBI in stick representation. The active site Lys^9^ was labelled (Yellow) and the intramolecular disulphide bond was present between Cys^6^ and Cys^16^ (Orange bond). (**F**) HPLC results of synthetic PE-BBI. **(G)** MALDI-ToF result of synthetic PE-BBI.

Skin secretions from *Pelophylax esculentus*, were fractionated by RP-HPLC using a C5 column (Fig. 1A). Samples of all 240 chromatographic fractions were subjected to MS/MS fragmentation. All of the fractions were subjected to a trypsin inhibition screening assay. Fraction #94 (arrowed) displayed strong trypsin inhibitory activity. Then the MS/MS fragmentation sequencing was applied to clarify the primary sequence of the mature peptide, PE-BBI (Fig. 1B).

### Molecular Cloning of PE-BBI Biosynthetic Precursor

The precursor cDNA open reading frame was obtained by two steps in molecular cloning. Briefly, the 3′-RACE reactions employed a nested universal primer (NUP) (supplied with the kit) and a sense primer (S: 5′-GGNGCNYTNAARGGNTGYTGGAGALKGCWT-3′) that is complementary to the N-terminal amino acid sequence. The product of 3′-RACE was further purified, then cloned using a pGEM-T vector system (Promega Corporation). Finally, the sample was sequenced by ABI 3100 sequencer. The specific antisense primer (AS: 5′-CCACATCAGATGACTTCCTCATCAT-3′), targeted to a conserved site within the 3′-non translated region of peptide precursor-encoding cDNA, was designed based on 3′-RACE products. Followed the same protocol, 5′-RACE was carried out. The full-length coding sequence was showed in Fig. 1C. The amino acid sequence and stick representation of PE-BBI was shown in Fig. 1D,E.

### Peptide Synthesis

The synthetic PE-BBI was detected by HPLC system in Fig. 1F. The purity of synthetic PE-BBI was higher than 95%. Subsequently, the synthetic replicates of PE-BBI was detected by MALDI-ToF. Three peaks were observed in Fig. 1G, 1812.907 Da for PE-BBI with intramolecular disulphide bridge, 1834.226 Da for PE-BBI with Sodium^+^ and 3623.609 Da for PE-BBI dimer, which generated from the intermolecular disulphide bond, the concentration of PE-BBI dimer was less than 2%. The synthetic replicates were applied in the following bio-assays.

### Trypsin Inhibitory Activity of PE-BBI

**Figure 2:**
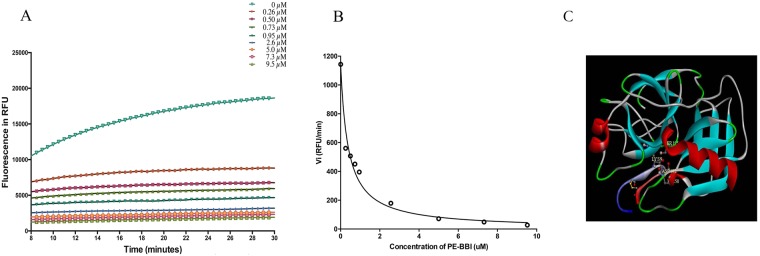
(**A**) Dose-dependent PE-BBI curves for trypsin proteolysis inhibitory assay. The x-axis represents time in minutes and the y-axis represents fluorescence intensity. (**B**) Morrison plots of PE-BBI, the initial rate (vi) values at each concentration of PE-BBI (0.1 to 100 µM). RFU/min (relative fluorescence units/min). (**C**) Possible interactions model between bovine trypsin protease and PE-BBI. The active site of trypsin (His^58^, Asp^102^, and Ser^195^) and the active site of PE-BBI (Lys^9^) were performed in stick representation. The disulphide bond of PE-BBI was showed in stick representation (Orange).

Synthetic replicates of PE-BBI was serially quantified for trypsin inhibitory activity in the range of 0.1–100 µM. PE-BBI showed a competitive reversible trypsin inhibitory activity. The dose-dependent hydrolysis of the fluorogenic substrates curve of PE-BBI were shown in Fig. 2A. The initial rates (v_i_) were calculated from Fig. 2A, and the initial rates were used to calculate the inhibition constants (K_i_) value by using ‘Morrison plots’ (Fig. 2B)^26^. Compared with some other BBI-type peptides such as HV-BBI and pLR-HL, PE-BBI was found to behave as a moderately potent trypsin inhibitor with a K_i_ value of 310 ± 72 nM. To further confirm the binding model between PE-BBI and bovine trypsin protease, we applied ZDock to simulate the binding model. From the simulation results, we can find that the active site (Lys^9^) of PE-BBI inserts into the reaction site (His^58^, Asp^102^, and Ser^195^) of bovine trypsin proteinase, thus blocking trypsin’s proteolytic activity. The simulation result shows PE-BBI might affect trypsin proteinase in the same way as reported BBI inhibitor peptide(s)/protein(s) (Fig. 2C).

### Myotropic Effects of PE-BBI

**Figure 3:**
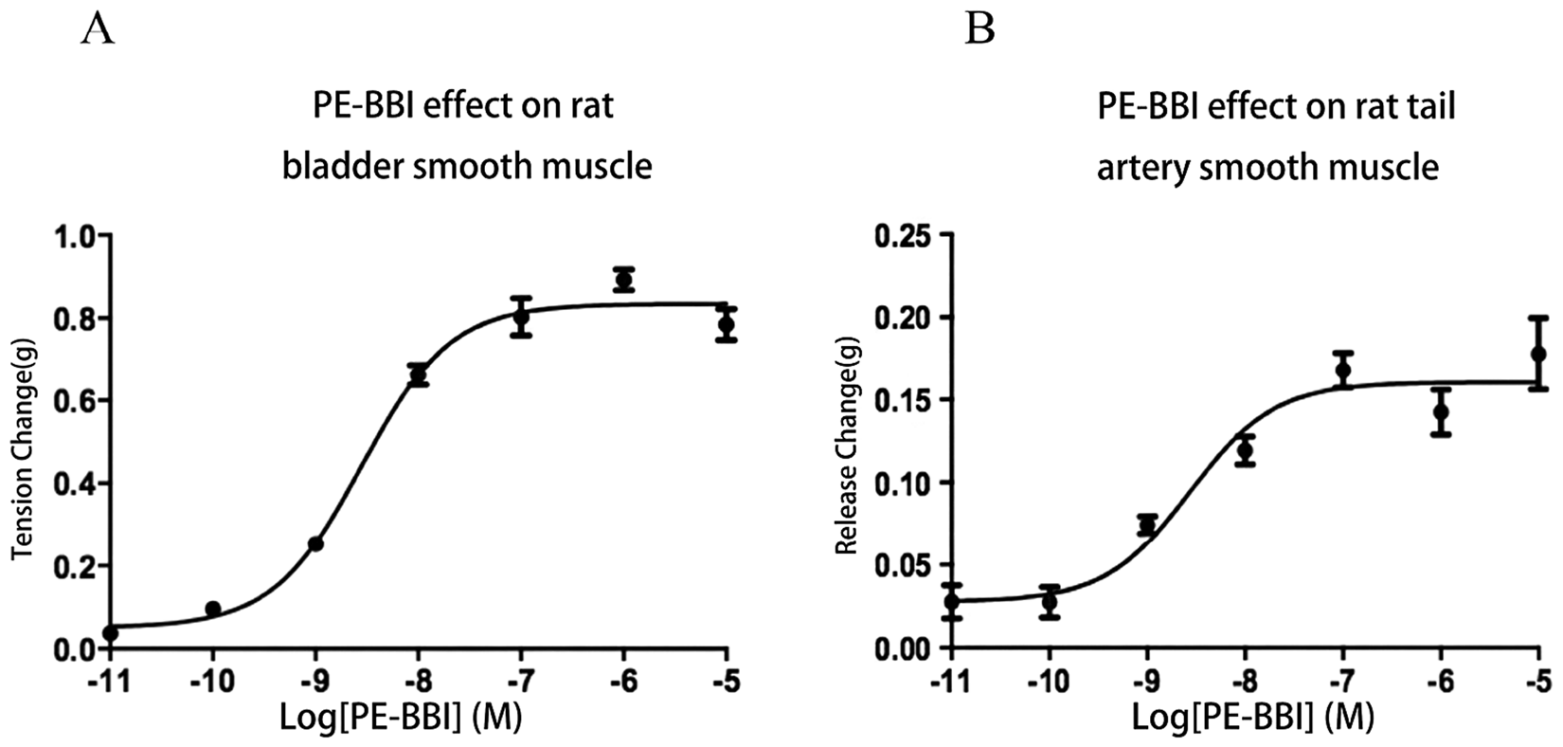
**(A**) Dose-response contraction effect of synthetic PE-BBI on rat bladder smooth muscle preparations. (**B**) Dose-response release of PE-BBI on rat-tail artery smooth muscle preparations. Data points in each panel represent the mean and SD of values obtained for 5 replicates.

Synthetic PE-BBI was found to be active in the rat tail artery and the bladder smooth muscle preparations (Fig. 3A,B). In the preparations of rat bladder smooth muscle, PE-BBI showed a dose-dependent contraction with an EC_50_ value of 14.25 nM (Fig. 3A). In preparations of rat artery, PE-BBI evoked a dose-dependent relaxation with an EC_50_ value of 8.79 nM (Fig. 3B).

### Antimicrobial Effects of PE-BBI

Antimicrobial activity of synthetic PE-BBI was assessed by determination of MIC assays using *E. coli*, *S. aureus*, and *C. albicans*. In this assay, PE-BBI didn’t show any obviously antibacterial activity even in the highest concentration (512 μM). The results illustrated that PE-BBI did not have antibacterial activity against bacterial and fungi (Data not showed).

### Anti-cancer Activity of PE-BBI

**Figure 4:**
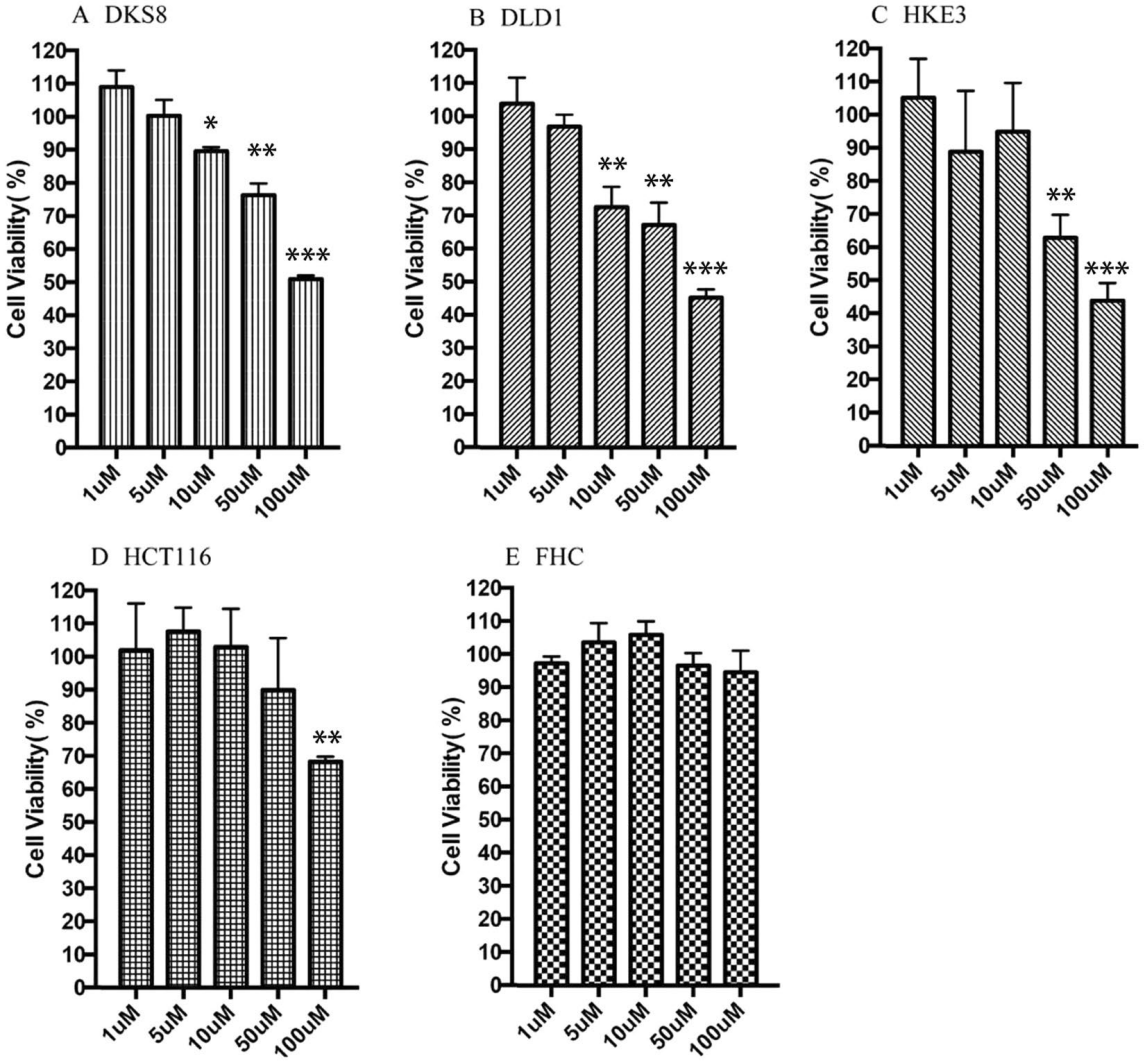
Dose-dependent anti-cancer activity of PE-BBI, ranging from 1 to 100 µM, against different human colorectal cancer cell lines **(A)** DKS8 **(B)** DLD-1 **(C)** HKE3 **(D)** HCT116 **(E)** FHC. The IC_50_ values against four cancer cell lines after 24 h incubation, were 50.1 µM, 9.8 µM, 35.4 µM, 50.2 µM, respectively. Meanwhile, PE-BBI didn’t show any cytotoxicity against human normal endothelial cell lines FHC. Data points in each panel represent the mean and SD of values obtained for 9 replicates. Significant differences were marked with asterisks (*P < 0.05; **P < 0,01; ***P < 0.001).

Previous studies supported the idea of the potential therapeutic use of BBI-type peptides as the anticancer therapy against human colon cancer. Thus, four human cancer cell lines including DLD-1, DKS8, HCT116, and HKE3 were used to examine the anti-proliferative effects of PE-BBI. Human colon endothelial cell line (FHC) was used to evaluate the inherent cytotoxicity of PE-BBI against normal human cells. PE-BBI showed selective cytotoxicity against the four different cancer cell lines and almost no cytotoxicity against FHC cells (Fig. 4A–E). The IC_50_ values against four cancer cell lines after 24 h incubation, were 50.1 µM, 9.8 µM, 35.4 µM, 50.2 µM, respectively. Meanwhile, PE-BBI didn’t show any cytotoxicity against human normal endothelial cell lines FHC.

### Molecular Docking Analysis Results of PE-BBI

**Figure 5:**
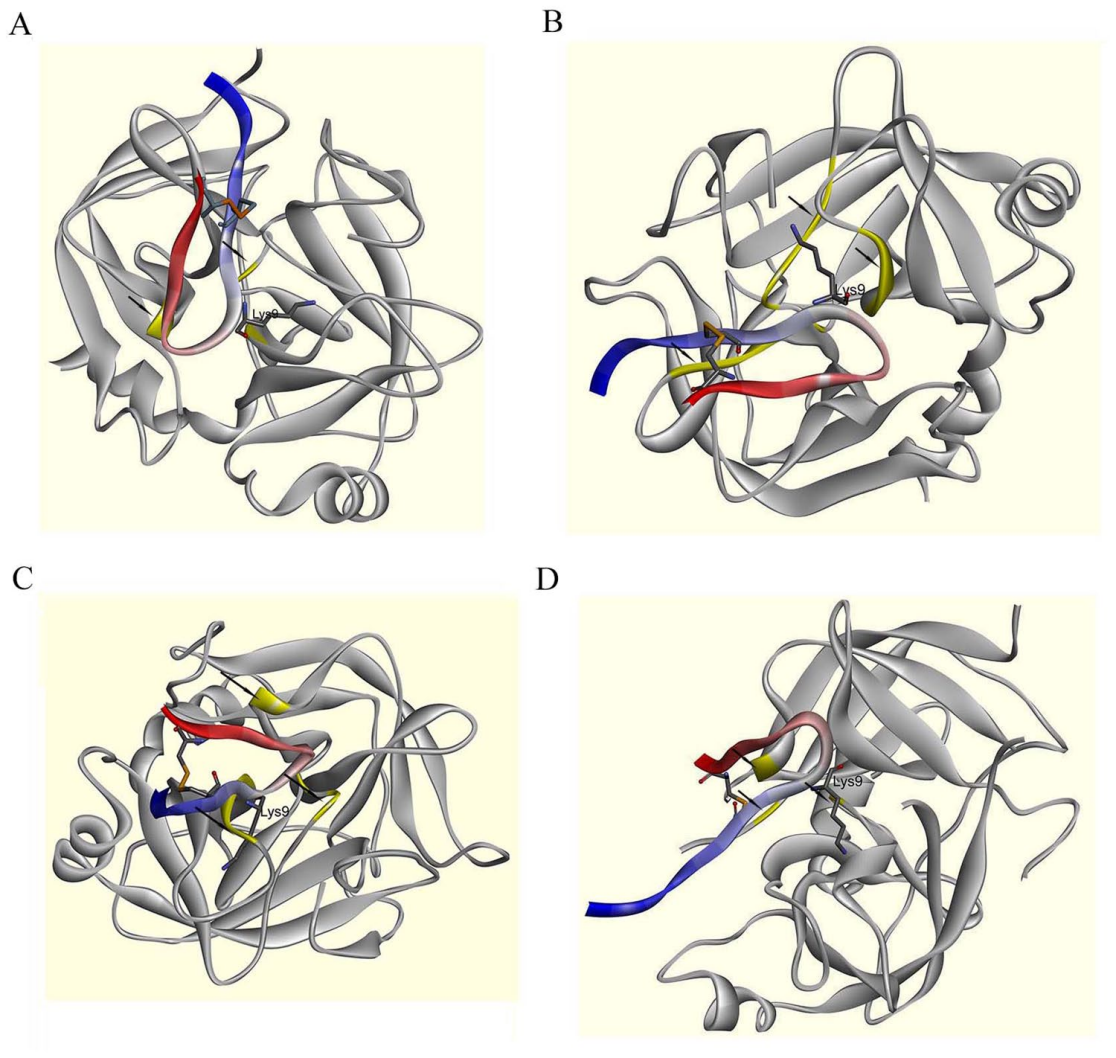
Structural characteristics of the protease-inhibitor docking output for PE-BBI and KLKs. The secondary structure of PE-BBI was labelled (N-terminal is blue and C-terminal is red), and the active site (Lys^9^) was shown in stick representation. The disulphide bond was showed in stick representation (Orange). The active site of KLKs were labelled with arrows and highlight (Yellow). (**A)** Possible interactions model between PE-BBI and KLK4. **(B)** Possible interactions model between PE-BBI and KLK6. **(C)** Possible interactions model between PE-BBI and KLK8. **(D)** Possible interactions model between PE-BBI and KLK10.

Molecular docking study was performed by BIOVIA Discovery Studio software to determine the putative docking of PE-BBI with KLKs. The visualized report of simulation was shown in Fig. 5. ZDock module of Discovery Studio was used for docking and scoring; then RDock was applied to optimize the binding results. The models optimized by RDock module were performed in Fig. 5A–D, respectively. The docking analysis revealed that PE-BBI is highly likely binding to the active site of KLKs. These results further confirmed our prediction of peptide binding mechanism. PE-BBI had high affinity to certain members of human KLKs protein, which could induce the inhibition of KLKs. Consequently, the inhibition of the KLKS would in turn also had implications for related cancer pathology processes.

## Discussion

**Figure 6:**
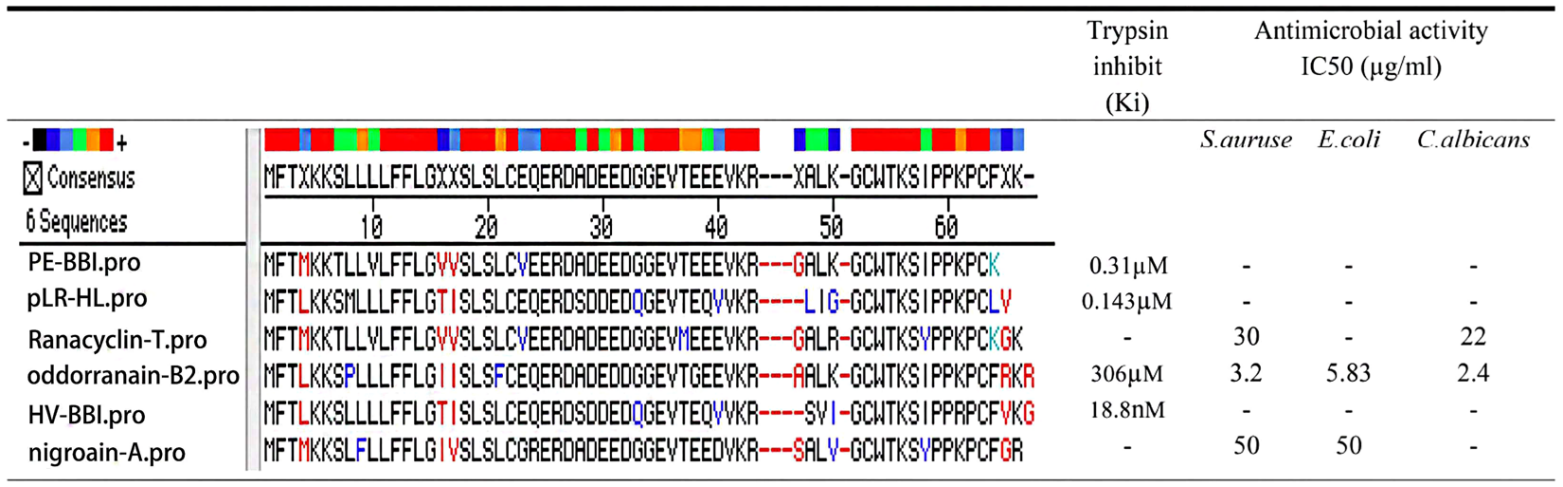
Comparison of different frog skin derived Bowman-Birk-type trypsin inhibitors^18,28–30,32^. “-” means no effect.

Amphibian skin secretions have long been known as complex cocktails of bioactive peptides. Several different classes of serine protease inhibitor peptides have been isolated and identified from frog skin secretions, including Bowman-Birk-like trypsin inhibitor peptides, which are classified according to their primary structure^9^. Skin secretion proteinase inhibitors are believed to be widespread in *Rana* frog species and their primary structure-based modifications can produce a series of analogues (shown in Fig. 6), such as HV-BBI and pLR-HL, two BBI trypsin inhibitor peptides with only trypsin inhibitory activity but no antimicrobial activity or anti-cancer activity^27,28^. Besides these peptides, some structurally similar peptides such as Nigroain-A and Ranacyclin-T, show broad antimicrobial activity but lack any trypsin inhibitory activity^29,30^. Interestingly, these peptides have highly similar primary structure but distinct bio-functions. Although many structurally similar trypsin inhibitor peptides have been found in the past years, until now, none of them showed any anticancer activity^28,30–32^. Leguminous BBIs have been identified with anti-cancer activity against human prostate, colon, breast, and skin cancer cell lines^33^. According to previous studies, a leguminous BBI showed *in vitro* and *in vivo* anti-tumour ability and without cytotoxicity in the dimethylhydrazine (DMH) induced colorectal tumour rat model^4,34^. Besides, BG-4, a novel proteinase peptide from *Momordica charantia* (bitter melon), induced apoptosis in HCT-116 and HT-29 colorectal cancer cells^20^. Another BBI peptide BTCI could form a stable complex with the 20S proteasome. In the complex form, BTCI can inhibit the trypsin-like proteolytic activity of the 20S proteasome, thereby inducing cytostatic and cytotoxic effects in MCF-7 breast cancer cells by induction of apoptosis^35^.

In this study, a novel short cyclic peptide PE-BBI was identified from the skin secretions of *Pelophylax esculentus*. It is the first time a frog skin BBI peptide was found to have the anticancer ability. The primary structure of PE-BBI contains the canonical disulphide-bridged loop between Cys^6^ and Cys^16^, which has been proved to be essential for their trypsin inhibitory capability^29^. Molecular docking analysis results also suggested that PE-BBI inactivates the trypsin proteinase through the combination between its cyclic part (Cys^6^ ~ Cys^16^) and the active site of trypsin proteinase. Synthetic PE-BBI demonstrated strong trypsin inhibitory activity (K_i_ = 310 ± 72 nM). Besides the proteinase inhibitory activity, it is interesting to see that PE-BBI also has a myotropic activity on isolated rat bladder and rat-tail artery smooth muscle on nano-/subnano-molar concentration. Through MTT assay, we also found that PE-BBI has considerable anticancer ability against a range of human colorectal cancer cell lines. Meanwhile, PE-BBI did not show any cytotoxicity against the normal human epithelial cell, FHC. Comparing the primary structure between PE-BBI and other frog skin-derived proteinase inhibitor peptides (Fig. 6)^18,28–30,32^, all of these peptides contained the same disulphide-bridged loop structure but differ entirely at the N-terminal and C-terminal extension amino acid residues. PE-BBI contains more lysine (K) and amidated C-terminal, which means PE-BBI has a more positive charge than pLR-HL and HV-BBI. From the previous research findings, we noticed that the cancer cell membrane contains more anionic lipids (e.g., phosphatidylserine) than noncancerous cells. Thus, the membrane of cancer cells is negative charged compared to the noncancerous cells. On the surface of noncancerous cells, due to zwitterionic phospholipids (e.g. phosphatidylcholine and sphingomyelin) occupies a large part, the surface of noncancerous cells is negligible or weak negative charged^36^. This hypothesis may explain why HV-BBI and pLR-HL peptide lack anti-cancer ability, due to the lack of the cell membrane binding capacity.

Regarding the mechanism of anti-cancer cytotoxicity of PE-BBI, several possibilities exist, based on some previous studies. Some researchers have suggested the anti-cancer ability of BBI is related to the suppression of the release of tumourigenesis promoting factors such as the superoxide anion radical and hydrogen peroxide^33,37–40^. Some other researchers propose that BBIs are able to inhibit some crucial proteolytic processes, which play key roles in carcinogenesis^31,33,41^.

Prognosis/diagnosis value of human tissue kallikrein-related peptidase (KLK) family members have been proved in many studies^21–24^. For instance, KLK4 dysregulation was detected in colon cancer and induced PAR1 signalling in caner. This study implies KLK4 may represent a novel pathway via PAR1 in colon tumourigenesis^42^. KLK6 mRNA overexpression evokes a poor prognosis in the colon cancer patient cohort^43^. Moreover, elevated KLK8 mRNA and protein level are detected in CRC tissue, which leads to an aggressive phenotype and poor prognosis^44^. The expression level of KLK10 is significantly higher in ovarian, pancreatic and colon cancer compared to normal tissues^23,45,46^. Previous studies have identified that trypsin and KLK4 shared significant structural and sequence similarity, and were both trypsin-like serine proteases. Thus, trypsin inhibitor would be able to inhibit KLK4 in addition to trypsin^47^. Swedberg *et al*. identified that SFTI-1 had high affinity to KLK4^47^. Thus, PE-BBI contains the homology active site region (-TK_P1_SIPP-) with SFTI-1, which means PE-BBI may represent similar affinity to KLK4. The docking results in the present study revealed the active regions of PE-BBI and KLK4 superimpose extremely well. Moreover, we also tested the binding between PE-BBI and other potential colon cancer biomarkers (KLK6, KLK8, and KLK10)^23,43–46^ and got the similar results as KLK4. The myotropic regulation effect of PE-BBI proved further evidence. KLK family is one of the key regulatory factors of tissue smooth muscles by regulating the kallikrein-kinin system^48^. The inhibition of KLKs would result in smooth muscle contraction/release. Thus, PE-BBI might inhibit cancer cell growth by blocking KLK proteins, which also affects the kallikrein-kinin system *in vivo*.

Overall, our study identified a novel multi-functional peptide PE-BBI and assigned its potential anticancer mechanism. Compared with leguminous derived BBIs, frog skin-derived BBI peptide PE-BBI has a more conserved primary structure with smaller molecule size, and is a potential anti-cancer lead compound. Further studies on the structure–function relationships and mechanisms of the anticancer ability of frog skin-derived BBI peptides may provide new candidates for peptide anti-cancer therapies.

## Methods and Materials

### Materials

*Pelophylax esculentus* (*n* = 5) were purchased from a local herpetological supplier. Trifluoroacetic acid (TFA), acetonitrile, trypsin from bovine pancreas and trypsin substrate (Gly-Gly-Arg-AMC, Sigma/Aldrich, Poole, Dorset, UK). Mueller–Hinton broth (MHB) (Sigma-Aldrich, St. Louis, MO, USA). DLD-1, DKS8, HCT116, HKE3 cell lines were cultured in Dulbecco’s Modified Eagle’s Medium F12 (DMEM F12) with 10% added FBS and 1% Penicillin-Streptomycin. FHC (ATCC-CRL-1831), and the cells were cultured in DMEM F12 medium (Gibco, Paisley, UK) containing 10% FBS, 10 ng/mL cholera toxin, 25 mM HEPES, 0.005 mg/mL insulin, 0.005 mg/mL transferrin, 100 ng/mL hydrocortisone, and 1% Penicillin-Streptomycin. DLD-1, HCT116, and FHC were supplied by American Type Culture Collection (ATCC, Teddington, Middlesex, UK). DKS8 and HKE3 were gifts from Prof. Senji Shirasawa’s Laboratory.

### Acquisition of *Pelophylax esculentus* Skin Secretion

The adult frogs (6–8 cm snout to vent length) were kept for 4 months prior to secretion harvesting. Transdermal electrical stimulation was used to harvest the skin secretions. This technique was first mentioned by Tyler and colleagues in 1992^49^. Briefly, the dorsal skin of frogs was stimulated by platinum electrodes (6 V DC; 4 ms pulse-width; 50 Hz) for two times, 20s per duration, then chilled deionised water was used to wash and collect the skin secretion before submerging into liquid nitrogen for lyophilise. Lyophilisate was stored at −20 °C prior to analysis. All of the above protocols were performed under the UK Animal (Scientific Procedures) Act 1986, project licence PPL 2694, issued by the Department of Health, Social Services and Public Safety, Northern Ireland. Procedures had been vetted by the IACUC of Queen’s University Belfast, and approved on 1 March 2011.

### Fractionation of Lyophilised *Pelophylax esculentus* Skin Secretion by Reverse Phase HPLC

The lyophilised skin secretion (5 mg) was dissolved in 0.5 mL of trifluoroacetic acid (TFA)/water (0.05/99.95, v/v). Then the solvent was centrifuged to collect the supernatant. The supernatant was analysed by using Thermoquest gradient system with an analytical column (Phenomenex C-5; 0.46 cm × 25 cm). The linear elution gradient (From 0.05/99.95 (v/v) TFA/water to 0.05/19.95/80.0 (v/v/v) TFA/water/acetonitrile) was applied to separate the peptides. The flow rate was 1 mL/min. The effluent absorbance was monitored at *λ*214 nm and 1 mL fractions were collected at minute intervals. After 240 minutes, all of the fractions were collected. The fractions (240 fractions in total) were separated, lyophilised and stored at −20 °C prior to analysis and screening.

### Structural Characterisation of PE-BBI

After trypsin inhibitory screening, fraction #94 which showed the highest trypsin inhibitory activity was further applied in the structural characterisation. The molecular weight of peptides in fraction #94 was detected by using MALDI-TOF mass spectrometry (Voyager DE instrument, Perspective Biosystems, MA, USA). The peptides with similar molecular weight with those mature peptides obtained from molecular cloning were subjected to LCQ Fleet electrospray ion-trap mass spectrometer (Thermo Scientific, San Jose, CA, USA) to clarify their primary structure. Then the mature peptide of PE-BBI was chemically-synthesized by solid phase peptide synthesis (SPPS) technology.

### Cloning of PE-BBI Precursor-Encoding cDNA

Lyophilised frog skin secretion (5 mg) was lysed by mRNA protection buffer by Dynal Biotec, UK. The polyadenylated mRNA was isolated by using magnetic oligo-dT beads as described by the manufacturer (Dynal Biotec, UK). To obtain the completed full-length trypsin inhibitor precursor encoding region, 5′- and 3′-rapid amplification of cDNA ends (RACE) procedures were applied using a SMART-RACE kit (Clontech, UK) followed manufacturer protocol^50^.

### Antimicrobial Assay

Gram-negative bacteria *Escherichia coli* (NCTC 10418); Gram-positive bacteria *Staphylococcus aureus* (NCTC 10788) and pathogenic yeast *Candida albicans* (NCYC 1467) were used in minimum inhibitory concentration (MIC) assays to investigate the board antimicrobial activity ability of synthetic PE-BBI. Microorganisms involved in MIC assays were cultured in MHB for 16–18 h. Then sub-cultured for 2~4 h to allow the microorganism to enter the logarithmic growth phase. Subsequently, proper density of bacterial (1 × 10^6^ colony forming units (cfu)/mL) and fungi (1 × 10^5^ cfu/mL) were seeded into 96 well plate. Different concentration ranged from 1 to 512 μM of PE-BBI were added into each well (6 replicates per concentration). The absorbance value at 550 nm was detected using a Synergy HT plate reader (Biotech, Minneapolis, MN, USA) after 18 h incubation in 37 °C incubator^50^.

### Myotropic Assay of PE-BBI

Female Wistar rats (250–300 g) were applied in this study and sacrificed followed the principle of institutional animal experimentation ethics and UK animal research guidelines. All of the procedures involved in this study was approved by IACUC of Queen’s University Belfast. Rat-tail artery and bladder were isolated and located in pre-chilled Kreb’s solution equilibrated with 95% O_2_, 5% CO_2_^51^. Isolated muscle strips (2–10 mm) were vertically suspended in a 2 mL organ bath containing 37 °C Kreb’s solution. The Kreb’s solution was constant refreshing by constant flowing (2 mL/min) during the assay. Potassium chloride (KCL, 60 mM) was used to test for viability of muscle strips after 20 min equilibration. After the smooth muscle strip were prepared, the synthetic replicates of PE-BBI (10^−11^ to 10^−5^ M) was used to quantify the myogenic potency on rat-tail artery and bladder smooth muscle. Between each concentration, the smooth muscle strip was washed 5 mins and re-equilibration for another 5 min. Each data point was repeated on at least five independent muscle strips. The tension change was amplified and recorded by a PowerLab System (AD Instruments Pty Ltd.)^51^.

Trypsin Inhibitor Assay of PE-BBITrypsin (0.1 µM in 1 mM HCL) and trypsin substrate (50 µM) were prepared prior to the assay. Trypsin inhibitory assay included positive group (10 µL trypsin, 180 µL substrate, 20 µL PBS), experiment group (10 µL trypsin, 180 µL substrate, 20 µL PE-BBI ranging from 0.1–100 µM) and control group (180 µL substrate, 30 µL PBS). In each well, the final volume was 210 µL. After 8 mins incubation, the fluorescent strength released from substrate hydrolysis was monitored continuously at 460 nm (excitation at 360 nm) in a CYTOFLUOR^®^ multi-well plate reader Series 4000 spectrofluorometer. Each sample was carried out in triplicate and repeated at least three times. The fluorescent strength values were used to calculate the initial rate (vi) and inhibition constants (K_i_)^32^.

### MTT Assay of PE-BBI

Synthetic replicates of PE-BBI were screened on the human colon cancer cell lines, DLD-1, DKS8, HCT116, and HKE3. Human colon endothelial cell FHC was used to evaluate the cytotoxicity of synthetic peptides on normal human cell line. All the cell lines were seeded with 5000 cells/well in 96 well plate. The seeded cells were incubated for 24 h and further starved for 12 h in prior of experiment, a series of PE-BBI concentrations (10^−7^–10^−4^ M) were prepared in related growth medium and added in the experiment group with 6 replicates. The same volume of PBS was added in the control group. After 24 h PE-BBI treatment, MTT assay was used followed the protocol described in detail previously^52^. Briefly, 10 µL of MTT (5 mg/mL) was added and incubated for 4 h. Then the supernatant was removed and 100 µL of DMSO was added to dissolve the purple formazan generated by live cells. The absorbance was measured at 570 nm by using EL808 TM Absorbance Microplate Reader (Biolise BioTek EL808, Winooski, VT, USA). The cell viability (%) of each concentrations were calculated by normalized to the control, respectively.

### Molecular Docking Simulation Analysis

Molecular docking simulation of PE-BBI and trypsin proteinase was carried out using BIOVIA Discovery Studio 2017 R2 (Biovia, San Diego, CA, USA). The purpose of molecular docking was simulated the predominant binding condition(s) between ligand peptide (PE-BBI) and receptor proteins (KLK family members) with known three-dimensional structure. The three-dimensional (3D) structure of bovine trypsin (PDB ID: 2O9Q), KLK4 (PDB ID: 4K8Y), KLK6 (PDB ID: 3VFE), KLK8 (PDB ID: 5MS4), and KLK10 (PDB ID: 5LPE) were obtained from the Protein Data Bank (https://www.rcsb.org/). Before docking, interior water molecules and ligands should be deleted to clear the docking candidates. The defaulted hided hydrogen atoms were added to both proteins. Subsequently, we used ZDock^53^, a rigid-body docking algorithm, to create the initial docking poses between PE-BBI and trypsin, KLK4, KLK6, KLK8, and KLK10. We applied the protocol with parameters as following: Angular Step Size as 15; RMSD cutoff as 6.0; Interface Cutoff as 9.0; Maximum Number of Clusters as 60. The ZDock protocol generated lots of docking poses ranked by the ZDock score. ZDock score was based on shape complementary to evaluate the reliability of docking poses. The top ranked poses were classified into different clusters^54,55^, and further refined by RDock^56^. RDock applied the Chemistry at Harvard Macromolecular Mechanics (CHARMm) force field to generate the energy minimized docking models. In this study, the top ranked ZDock poses were optimized by RDock then further applied in the molecular docking^57^.

### Statistical Analysis

Statistical analysis was performed using GraphPad Prism 6 software. P values were calculated by Student’s t-test from the mean values of the indicated data. Significant differences were demonstrated with asterisks (*P < 0.05; **P < 0,01; ***P < 0.001).
